# Association between absolute volumes of lung spared from low-dose irradiation and radiation-induced lung injury after intensity-modulated radiotherapy in lung cancer: a retrospective analysis

**DOI:** 10.1093/jrr/rrv057

**Published:** 2015-10-09

**Authors:** Jinmei Chen, Jinsheng Hong, Xi Zou, Wenlong Lv, Feibao Guo, Hualan Hong, Weijian Zhang

**Affiliations:** 1 Department of Radiation Oncology, First Affiliated Hospital, Fujian Medical University, No. 20 Chazhong Road, Taijiang District, Fuzhou City, Fujian Province, China; 2 Key Laboratory of Radiation Biology, Fujian Medical University, Fujian Province University, No. 20 Chazhong Road, Taijiang District, Fuzhou City, Fujian Province, China

**Keywords:** lung cancer, radiation-induced lung injury, intensity-modulated radiotherapy, low-dose irradiation volume

## Abstract

The aim of this study was to investigate the association between absolute volumes of lung spared from low-dose irradiation and radiation-induced lung injury (RILI) after intensity-modulated radiotherapy (IMRT) for lung cancer. The normal lung relative volumes receiving greater than 5, 10, 20 and 30 Gy (V5–30) mean lung dose (MLD), and absolute volumes spared from greater than 5, 10, 20 and 30 Gy (AVS5–30) for the bilateral and ipsilateral lungs of 83 patients were recorded. Any association of clinical factors and dose–volume parameters with Grade ≥2 RILI was analyzed. The median follow-up was 12.3 months; 18 (21.7%) cases of Grade 2 RILI, seven (8.4%) of Grade 3 and two (2.4%) of Grade 4 were observed. Univariate analysis revealed the located lobe of the primary tumor. V5, V10, V20, MLD of the ipsilateral lung, V5, V10, V20, V30 and MLD of the bilateral lung, and AVS5 and AVS10 of the ipsilateral lung were associated with Grade ≥2 RILI (*P* < 0.05). Multivariate analysis indicated AVS5 of the ipsilateral lung was prognostic for Grade ≥2 RILI (*P* = 0.010, OR = 0.272, 95% CI: 0.102–0.729). Receiver operating characteristic curves indicated Grade ≥2 RILI could be predicted using AVS5 of the ipsilateral lung (area under curve, 0.668; cutoff value, 564.9 cm^3^; sensitivity, 60.7%; specificity, 70.4%). The incidence of Grade ≥2 RILI was significantly lower with AVS5 of the ipsilateral lung ≥564.9 cm^3^ than with AVS5 < 564.9 cm^3^ (*P* = 0.008). Low-dose irradiation relative volumes and MLD of the bilateral or ipsilateral lung were associated with Grade ≥2 RILI, and AVS5 of the ipsilateral lung was prognostic for Grade ≥2 RILI for lung cancer after IMRT.

## INTRODUCTION

Radiation-induced lung injury (RILI) is the major dose-limiting toxicity after radiotherapy in lung cancer. In clinical practice, RILI can be relieved to a degree by hormone therapy; however, medium or severe RILI still significantly affects the quality of life of patients, and may even lead to death [1, 2]. In recent years, because of high-conformity dose distribution with protection of surrounding tissues, the application of intensity-modulated radiotherapy (IMRT) for lung cancer has made it possible to increase the radiation dose; this has resulted in better treatment outcomes compared with 3D conformal radiotherapy (3D-CRT). However, the occurrence of RILI cannot yet be disregarded [3, 4]. Studies have reported close associations of the normal lung relative volume of low-dose irradiation (Vx) and mean lung dose (MLD) of the bilateral lung with the occurrence of RILI [3, 4]. Clinically, both of these dosimetric parameters are currently carefully controlled in order to reduce the risk of RILI. However, RILI still occurs at a high rate, even when these parameters are strictly controlled [3, 4]. Therefore, it is clinically urgent to further explore other factors that contribute to the risk of RILI so that IMRT treatment planning can be improved. Recently, two studies on patients receiving 3D-CRT found that the normal lung absolute volume spared from greater than a particular level of low-dose irradiation (AVSx) of the bilateral lung and Vx of the ipsilateral lung should also be controlled during radiotherapy planning [5, 6]. However, it is not known whether these findings also apply in patients receiving IMRT. Furthermore, is the AVSx of the ipsilateral lung associated with the risk of RILI after IMRT in patients with lung cancer? In this research, we explored risk factors for RILI after IMRT in patients with lung cancer by examining the relationship of dose–volume parameters such as the AVSx of the ipsilateral lung and other clinical factors with the occurrence of RILI.

## MATERIALS AND METHODS

### Patients and clinicopathological features

A retrospective analysis was performed on the medical records of 119 patients with lung cancer treated between June 2009 and September 2013. The inclusion criteria were patients (i) with pathologically confirmed lung cancer, (ii) who were receiving chest radiotherapy for the first time, (iii) who had no treatment interruptions of longer than 5 days during radiotherapy, and (iv) who survived at least 6 months from the beginning of radiotherapy. Patients were excluded if (i) the follow-up time was less than 6 months or (ii) they received a second round of chest radiotherapy after recurrence. Of the 119 patients, 17 were excluded because they survived for less than 6 months, 8 were excluded because they received a second round of chest radiotherapy after recurrence, 6 were lost to follow-up and 5 were followed-up for less than 6 months.

A total of 83 patients qualified and were included in the study. The median age of the patients was 61 years old (range, 32 to 84); 75 patients were male and eight were female. Of the 83 patients, 33 had squamous cell carcinoma, 22 had adenocarcinoma, 20 had small cell carcinoma and 8 had other pathological types of lung cancer. According to the 7th edition of the AJCC staging system (2010), 10 patients had Stage I disease; 14, Stage II; 52, Stage III; and 7, Stage IV. Forty-six patients had central carcinoma and 37 had peripheral carcinoma; 51 patients had a primary tumor in the upper lobe and 32 in the middle or lower lobe; 56 patients were smokers/former smokers and 27 were non-smokers; 18 patients had undergone one or more pulmonary surgeries and 65 had not undergone surgery; 48 patients had underlying lung disease (including emphysema, chronic bronchitis, allergic asthma, pulmonary bullae, etc.) and 35 had no underlying lung disease.

### Treatment planning

The median volume of the primary tumor was 64.2 cm^3^ (4.2–428.5 cm^3^), the median fractionated dose was 2.1 Gy (2.0–2.5 Gy) and the median equivalent dose at 2 Gy per fraction (EQD2) was 59.9 Gy (42.4–73.5 Gy). The dose limits for the organs at risk were as follows: bilateral lung, define (V20) ≤ 37% and MLD ≤ 20 Gy; spinal cord, maximum dose ≤ 45 Gy; heart, V40 ≤ 40%; and esophagus, V50 ≤ 50%. Lung was defined to exclude the gross target volume (GTV). The collapsed cone convolution was chosen for the treatment planning. All patients received 5-beam 6-MV X-ray IMRT, delivered by a linear accelerator (Clinac 600C/D; Varian Medical Systems, Palo Alto, CA, USA), with one fraction per day and five fractions per week. Seventy-two patients received induction chemotherapy; 11 patients did not receive induction chemotherapy. Patients with non–small cell carcinoma received a paclitaxel, docetaxel or gemcitabine plus platinum regimen; patients with small cell carcinoma received a VP-16 plus cisplatin regimen. Twelve patients received concurrent chemotherapy; of these cases, patients with non–small cell carcinoma received single agent paclitaxel or docetaxel chemotherapy and patients with small cell carcinoma received a VP-16 plus cisplatin regimen. Forty-four patients received adjuvant chemotherapy; of these cases, patients with non–small cell carcinoma received a regimen with paclitaxel, docetaxel or gemcitabine plus platinum or not, and patients with small cell carcinoma received a VP-16 plus cisplatin regimen.

### Dosimetric indicators

A dose–volume histogram (DVH) related to the treatment planning system (TPS, Pinnacle3; Philips Medical Systems, Andover MA) was used to calculate the normal lung volume receiving greater than 5, 10, 20 and 30 Gy (V5, V10, V20, V30), MLD, and absolute volumes spared from greater than 5, 10, 20 and 30 Gy (AVS5, AVS10, AVS20, AVS30) of the bilateral lung and the ipsilateral lung.

### RILI follow-up and evaluation

The symptoms of the patients were reviewed weekly during radiotherapy. The symptoms and the lung CT images were reviewed 3–4 weeks after completion of treatment, every 2–3 months in the first two years, and at 6-month intervals during the third to fifth year. RILI was assessed and graded according to the RTOG criteria [7].

### Statistical analysis

Chi-squared tests or Student's *t*-tests were used for univariate analysis of the association of clinical factors or dose–volume parameters with the occurrence of RILI. Pearson's correlation analysis was performed to examine the colinearity of the AVS5 of ipsilateral lung and quantitative indicators. Student's *t*-test analysis was performed to examine the correlation between the AVS5 of the ipsilateral lung and qualitative indicators. Logistic regression analysis was used to identify the factors associated with RILI; receiver operating characteristic (ROC) curve analysis was used to assess the sensitivity and specificity of the significant factors for predicting RILI. Chi-squared tests were used to examine the rates of RILI occurrence. *P* < 0.05 was considered significant for all analysis. The software of SPSS version 17.0 (SPSS Inc., Chicago, IL, USA) was used for statistical analysis.

## RESULTS

### Occurrence of RILI

The median follow-up time for the entire cohort was 12.3 months (6.1–52.0 months). Of the 83 patients with lung cancer who received IMRT, 56 patients (67.5%) developed Grade ≤ 1 RILI, 18 patients (21.7%) developed Grade 2 RILI, 7 patients (8.4%) developed Grade 3 RILI, 2 patients (2.4%) developed Grade 4 RILI, and no patients developed Grade 5 RILI.

### Univariate and multivariate analysis of the factors affecting the occurrence of Grade ≥2 RILI

Univariate analysis showed that the lobe in which the primary tumor was located was associated with the occurrence of Grade ≥2 RILI (*P* < 0.05; Table 1). Additionally, there were significant associations between the occurrence of Grade ≥2 RILI and the V5, V10, V20 and MLD of the ipsilateral lung, the V5, V10, V20, V30 and MLD of the bilateral lung, and the AVS5 and AVS10 of the ipsilateral lung (all *P* < 0.05; Table 2).

**Table 1.** Univariate analysis of the association of clinical factors with RILI in 83 patients with lung cancer receiving IMRT

Factor	Grade ≤1 RILI	Grade ≥2 RILI	*P-*value
Sex			0.752
Male	51	24	
Female	5	3	
Pathological type			0.464
Squamous cell carcinoma	21	12	
Adenocarcinoma	13	9	
Small-cell lung carcinoma	16	4	
Other	6	2	
Location of primary tumor			0.650
Central	32	14	
Peripheral	24	13	
Lobe of primary tumor			0.027
Upper	39	12	
Middle/Lower	17	15	
Smoker			0.914
Yes	38	18	
No	18	9	
Ever received pulmonary surgery			0.627
Yes	13	5	
No	43	22	
Underlying lung disease			0.086
Yes	36	12	
No	20	15	
Induction chemotherapy			0.689
Yes	48	24	
No	8	3	
Concurrent chemotherapy			0.163
Yes	6	6	
No	50	21	
Adjuvant chemotherapy			0.428
Yes	28	16	
No	28	11	

RILI = radiation-induced lung injury.

**Table 2.** Association between dose–volume parameters and RILI in 83 patients with lung cancer receiving IMRT

Dose–volume parameter	Grade ≤1 RILI	Grade ≥2 RILI	*P-*Value
V5 of ipsilateral lung (%)*	61.4 ± 17.6	70.1 ± 12.8	0.001
V10 of ipsilateral lung (%)*	52.7 ± 14.9	61.6 ± 11.0	0.003
V20 of ipsilateral lung (%)*	34.5 ± 7.8	39.5 ± 7.5	0.007
V30 of ipsilateral lung (%)	23.9 ± 8.9	26.7 ± 7.4	0.158
MLD of ipsilateral lung (Gy)*	17.5 ± 4.4	20.4 ± 3.3	0.003
AVS5 of ipsilateral lung (cm^3^)*	665.2 ± 370.1	450.2 ± 275.3	0.004
AVS10 of ipsilateral lung (cm^3^)*	809.3 ± 374.2	622.8 ± 282.8	0.025
AVS20 of ipsilateral lung (cm^3^)	1106.4 ± 396.0	948.2 ± 301.5	0.070
AVS30 of ipsilateral lung (cm^3^)	1291.2 ± 484.1	1143.8 ± 336.3	0.112
V5 of bilateral lung (%)*	53.7 ± 15.5	62.0 ± 12.8	0.018
V10 of bilateral lung (%)*	40.0 ± 11.9	45.9 ± 11.4	0.034
V20 of bilateral lung (%)*	21.2 ± 5.4	24.6 ± 6.5	0.013
V30 of bilateral lung (%)*	12.1 ± 3.3	14.9 ± 5.1	0.012
MLD of bilateral lung (Gy)*	12.1 ± 3.6	14.2 ± 2.9	0.009
AVS5 of bilateral lung (cm^3^)	1713.0 ± 728.7	1400.3 ± 710.2	0.068
AVS10 of bilateral lung (cm^3^)	2200.9 ± 712.1	1915.5 ± 799.5	0.104
AVS20 of bilateral lung (cm^3^)	2875.0 ± 739.6	2614.1 ± 809.1	0.148
AVS30 of bilateral lung (cm^3^)	3212.7 ± 830.9	2940.8 ± 848.9	0.169
EQD2 (Gy)	58.6 ± 7.3	58.6 ± 6.5	0.987
Primary tumor volume (cm^3^)	86.5 ± 88.8	99.3 ± 69.1	0.512

RILI = radiation-induced lung injury, Vx = relative volume of normal lung irradiated with a dose > X Gy, MLD = mean lung dose, AVSx = absolute volume of normal lung spared from irradiation at a dose > X Gy, EQD2 = equivalent dose at 2 Gy/fraction. *Statistically significant association.

Collinearity analysis of the factors that were demonstrated to be related to the occurrence of Grade ≥2 RILI by univariate analysis, showed that: the AVS5 of the ipsilateral lung was significantly associated with the AVS10, V5, V10, V20 and MLD of the ipsilateral lung, and the V5, V10, V20 and MLD of the bilateral lung (*P* ≤ 0.001), yet no significant relationship was observed between the AVS5 of the ipsilateral lung and the V30 of the bilateral lung (*P* = 0.094, Table 3). Additionally, grouped *t*-tests indicated a significant relationship between the lobe in which the primary tumor was located and the AVS5 of the ipsilateral lung (*t* = 4.842, *P* < 0.001).

**Table 3.** Correlation of the AVS5 of the ipsilateral lung with other dose–volume parameters

Dose–volume parameter	Correlation coefficient	*P-*value
AVS10 of ipsilateral lung*	0.979	<0.001
V5 of ipsilateral lung*	−0.800	<0.001
V10 of ipsilateral lung*	−0.767	<0.001
V20 of ipsilateral lung*	−0.470	<0.001
MLD of ipsilateral lung*	−0.504	<0.001
V5 of bilateral lung*	−0.690	<0.001
V10 of bilateral lung*	−0.566	<0.001
V20 of bilateral lung*	−0.292	<0.001
V30 of bilateral lung	−0.185	0.094
MLD of bilateral lung*	−0.360	0.001

AVSx = absolute volume of normal lung spared from receiving irradiation with a dose > X Gy, Vx = relative volume of normal lung irradiated with a dose > X Gy, MLD = mean lung dose. *Statistically significant correlation.

Based on the collinearity analysis above, logistic regression analysis was performed using four factors (AVS5 of the ipsilateral lung; V30 of the bilateral lung; presence or absence of underlying lung disease; receiving concurrent chemotherapy or not) as covariables and Grade ≥2 RILI as a dependent variable. The AVS5 of the ipsilateral lung was an independent prognostic factor for the occurrence of Grade ≥2 RILI (*P* = 0.010, OR = 0.272, 95% CI: 0.102–0.729; Table 4).

**Table 4.** Multivariate analysis of the association between dose–volume parameters and Grade ≥2 RILI in 83 patients with lung cancer receiving IMRT

Covariable	*B*	*Wald*	OR Value (95% CI)	*P-*value
AVS5 of ipsilateral lung	−1.300	6.696	0.272 (0.102–0.729)	0.010
V30 of bilateral lung	0.715	2.039	2.044 (0.766–5.452)	0.153
Underlying lung disease	0.608	1.394	1.836 (0.670–5.032)	0.238
Concurrent chemotherapy	−0.703	1.017	0.495 (0.126–1.941)	0.313
Constant	1.154	2.434	3.170	0.119

AVS5 = absolute volume of normal lung spared from irradiation at a dose > 5 Gy, V30 = relative volume of normal lung receiving irradiation at a dose > 30 Gy.

### Prognostic value of the AVS5 of the ipsilateral lung

The occurrence of Grade ≥2 RILI in lung cancer patients receiving IMRT could be predicted with a sensitivity of 60.7% and a specificity of 70.4% on the basis of the AVS5 of the ipsilateral lung using an area under the ROC curve of 0.668 and a cut-off value of 564.9 cm^3^. Of the 83 patients in this study, 41 had an AVS5 < 564.9 cm^3^ for the ipsilateral lung, among whom 19 patients (46.3%) had Grade ≥2 RILI, and 42 patients had an AVS5 of the ipsilateral lung ≥564.9 cm^3^, among whom 8 (19.0%) had Grade ≥2 RILI; the difference in the rate of Grade ≥2 RILI between these groups was statistically significant (χ^2^ = 7.042, *P* = 0.008).

## DISCUSSION

The rates of occurrence of Grade ≥2 and Grade ≥3 RILI among the patients with lung cancer receiving IMRT in this study were 32.5% and 10.8%, respectively. This analysis indicated that the occurrence of Grade ≥2 RILI was not only associated with the MLD and Vx of low dose of the ipsilateral lung or bilateral lung, but was also closely associated with the AVS5 of the ipsilateral lung. The AVS5 of the ipsilateral lung was prognostic for the occurrence of Grade ≥2 RILI after IMRT in this cohort of patients with lung cancer. Additionally, the rate of occurrence of Grade ≥2 RILI was <20% in patients with an AVS5 of the ipsilateral lung ≥564.9 cm^3^.

The rate of occurrence of RILI after IMRT in patients with lung cancer remains high, even though IMRT can ensure a high-conformity dose distribution for the target volume and thereby better protect the normal tissues and organs at risk. Previous studies indicated that IMRT improved the survival of patients with non–small cell lung cancer compared with 3D-CRT; however, the rate of occurrence of Grade ≥3 therapy-associated pneumonia at 6 months after radiotherapy was 11% and that of Grade 1 pulmonary fibrosis was as high as 86% at 18 months after radiotherapy [3], with a rate of Grade 2 radiation pneumonitis of up to 23% in other reports [8]. Of the 20 patients with small cell lung cancer included in this study who received lower dose radiotherapy, 32.5% developed Grade ≥2 RILI and 10.8% developed Grade ≥3 RILI.

A number of studies have indicated that the V20 and MLD of the bilateral lung are associated with the occurrence of RILI [2, 9–13, 14]; yet these conclusions are mainly based on data from patients receiving 3D-CRT or patients receiving both 3D-CRT and IMRT. Compared with 3D-CRT, IMRT results in a larger lung volume of low-dose irradiation, thus reducing the V10 and V20, yet increasing V5 [15]. It has already been reported that the V5 of the ipsilateral lung and the bilateral lung were major factors associated with the occurrence of Grade ≥3 RILI in patients with non–small cell lung cancer receiving 3D-CRT [16, 17]. Analysis of data on patients receiving IMRT in our study also suggests that Grade ≥2 RILI was not only associated with the V20 and MLD of the bilateral lung and the ipsilateral lung, but also related to the V5 and V10 of the bilateral lung and the ipsilateral lung. In addition, our results also showed a trend in the relationship between the occurrence of RILI of Grade ≥2 and the Vx of the ipsilateral lung such that the lower the X value, the smaller the *P*-value (indicating more significance). Fatal radiation pneumonia may occur if a large volume of normal lung tissue is irradiated, even with a dose as low as 5 Gy [18]. Therefore, the capacity of the low-dose irradiation relative volumes of the bilateral lung and the ipsilateral lung to affect the occurrence of RILI after IMRT should not be ignored and warrants further investigation.

Since the tumor volume and lung volume vary between patients, there were significant differences in the normal lung volume of the bilateral lung, and especially of the ipsilateral lung, between patients. For example, the normal volume of the bilateral lung of the patients included in this study ranged from 1614.7–5982.9 cm^3^ and the normal volume of the ipsilateral lung ranged from 536.8–3266.7 cm^3^. One patient in this study had a normal lung volume of the ipsilateral lung of 536.8 cm^3^, with a V5 for the ipsilateral lung of 70.9%, a V5 for the bilateral lung of 50.2%, V20 of 17.8%, V30 of 11.5% and MLD of 11.5 Gy. All of these values are significantly lower than the corresponding threshold values recommended in related studies [19], yet this patient suffered Grade 3 acute radiation pneumonia. It is obvious that individual differences in the normal lung volume have been neglected when evaluating the radiotherapy plan by calculating the Vx. This raised the question of whether there is an association between the occurrence of RILI and the low-dose irradiation absolute volume of the normal lung, especially the AVSx values of the ipsilateral lung. This speculation was confirmed in this study: the ipsilateral AVS5, correlated significantly with the V5 to V20 values of the bilateral lung and the ipsilateral lung, was significantly associated with occurrence of Grade ≥2 RILI in the 83 patients with lung cancer receiving IMRT. However, no relationship was observed between the AVSx of the bilateral lung and the occurrence of RILI. In contrast to our result, Jenkins' analyses of a cohort of patients with lung cancer treated using 2–3-beam 3D-CRT and continuous accelerated hyperfractionated radiotherapy showed that AVS5, AVS10 and AVS15 of the bilateral lung was significant correlated with the risk of RILI [5, 6]. Since the patients reviewed in this study underwent IMRT with conventional fractionation, further research is required to determine if these discrepancies were due to the different therapy models and techniques in each study.

ROC curves were used to examine the capacity of the AVS5 of the ipsilateral lung for predicting the occurrence of Grade ≥2 RILI. When the area under the ROC curve was 0.668 using a cut-off value of 564.9 cm^3^, the AVS5 of the ipsilateral lung had a sensitivity of 60.7% and a specificity of 70.4% for predicting Grade ≥2 RILI. This area under the ROC curve is similar to that of the V5, V13, V20, V30 and MLD for predicting RILI reported in other studies [20–22]. Hence, the AVS5 of the ipsilateral lung cannot adequately predict the occurrence of RILI. It has been suggested that more than one dose–volume parameter from the DVH needs to be analyzed to accurately predict the risk of RILI in the clinic [23]. When creating IMRT treatment plans for lung cancer, the V5, V20 and MLD of the bilateral lung and the ipsilateral lung should be comprehensively evaluated, and special attention should be paid to the AVS5 of the ipsilateral lung if there is a small volume of normal lung on the involved side.

The patients included in this study underwent IMRT and conventional fractionated therapy. Our analysis demonstrated that specific dose–volume parameters were closely associated with the occurrence of RILI in patients treated with IMRT, and that the low-dose irradiation absolute volumes of the ipsilateral lung were closely associated with the occurrence of RILI. This information may help to optimize IMRT treatment planning for lung cancer. However, this study was a retrospective analysis; therefore, prospective studies and analyses of larger cohorts of patients treated at different institutions are needed to verify the accuracy of these results.

In conclusion, low-dose irradiation volumes and the MLD of the bilateral lung and the ipsilateral lung are associated with the risk of Grade ≥2 RILI. Additionally, the AVS5 of the ipsilateral lung was a prognostic factor for Grade ≥2 RILI, albeit with limited prognostic value. Additional dose–volume parameters need to be comprehensively evaluated in order to improve IMRT treatment planning and reduce the occurrence of RILI in patients with lung cancer.

## FUNDING

This work was supported by Grants from Natural Science Foundation of Fujian Province of China [2012J01338], Medical Innovation Research of Fujian Province of China [2012-CX-20], and Youth Scientific Research of Health Department of Fujian Province of China [2013-1-30]. Funding to pay the Open Access publication charges for this article was provided by Grants from Natural Science Foundation of Fujian Province of China [2012J01338], Medical Innovation Research of Fujian Province of China [2012-CX-20], and Youth Scientific Research of Health Department of Fujian Province of China [2013-1-30].
